# *Announcement*: Fungal Disease Awareness Week — August 14–18, 2017

**DOI:** 10.15585/mmwr.mm6631a6

**Published:** 2017-08-11

In 2017, CDC initiated a national observance, Fungal Disease Awareness Week, to increase awareness about fungal diseases, which can cause severe illness but frequently go undiagnosed. Awareness is one of the most important means to reduce delays in diagnosis and treatment, which can lead to better health outcomes and save lives.

The theme of this year’s observance is “Think Fungus,” and aims to encourage the public and clinicians to consider the possibility of a fungal infection if a patient’s symptoms are not improving with treatment. There are many types of fungal diseases. Immunocompromised persons are more likely to acquire serious fungal diseases, but some types of fungal infections occur in otherwise healthy persons.

Fungal diseases are an increasing problem worldwide, although the exact prevalence is difficult to quantify (*1*). In the United States, coccidioidomycosis (often called “Valley fever”) is particularly concerning; although approximately 10,000 cases are reported each year, it is likely that many more cases go undiagnosed, with an estimated 150,000 infections annually (*2*). This issue of *MMWR* includes a report on a substantial increase in coccidioidomycosis cases in California in 2016 (*3*). *Candida*, a common cause of mucosal and skin infections, is an important cause of bloodstream infections in hospitalized patients (*4*). Antifungal resistance is a growing public health problem, particularly in *Candida* and *Aspergillus* infections,* compounded by the recent emergence of *Candida auris*, a multidrug-resistant yeast that spreads in health care facilities (*5*). Resistant infections lead to longer hospital stays, higher medical costs, and more deaths. Globally, cryptococcal meningitis, histoplasmosis, and *Pneumocystis* pneumonia remain important causes of death in patients with human immunodeficiency virus infections and acquired immunodeficiency syndrome. Additional information about Fungal Disease Awareness Week is available at https://www.cdc.gov/fungal/awareness-week.html.

## Footnotes

* https://www.cdc.gov/drugresistance/threat-report-2013/index.html.
